# The History and Capabilities of Herbal Simples

**Published:** 1890-10-18

**Authors:** W. F. Fernie


### The History and Capabilities of Herbal Simples.

By W. T. Fernie, M.D.

### XXI.—CLOVER.

Common clover, or trefoil, was used at the festivals of the ancient Greeks. Furthermore, they thought it endowed with weatherwise properties. Pliny writeth and setteth it down for certain that the leaves of the common trefoil do tremble and stand right up against the coming of a storm or tempest. "The leaves do start up as if afraid of an assault when tempestuous weather is at hand." In this country we possess about twenty species of the trefoil, all of which contract their leaves when stormy weather is at hand. They have therefore been termed the husbandman's barometer. Our meadow clover (*trifolium pratense*), called by some cocks-heads, is known to children as suckles, or honeysuckles, because of the abundance of honey in the long tubes of its corollæ. A syrup is made from the flowers, which has a trustworthy reputation for curing whooping-cough, and of which a teaspoonful may be taken three or four times in the day. An extract of red clover is said to have the power of healing scrofulous sores and of curing cancer. The *New York Tribune*, of September, 1884, relates a case of indisputable cancer of the breast, of six years' standing, with an open fetid sore which had penetrated an intercostal space, being radically healed by an internal use of the extract of red clover. Again some good women lay great stress on a decoction of the purple and the white meadow trefoils for curing cutaneous outbreaks in children. Any four-leaved clover, through being in the form of a cross, is thought to give the person who carries it the power of detecting evil spirits, and the privilege of good luck at play.

"With a four-leaved clover, a double-leaved ash, and a green-topped seave,
You may go before the Queen's daughter without asking her leave."

An old Herbal says: "The meadow trefoil (especially that with the black half-moon on the leaf), stamped with a little honey, takes away the pin and web of the eies, if it be strained and dropped therein." A conventional trefoil is figured on our coins, both Irish and English; whilst the Irish people take the creeping trefoil as their national badge. It represents their much-beloved "Shamrock so green"; though some think the wood sorrel is a better substitute. Either species, from the triple arrangement of its leaves, has long been regarded as noisome to witches, and the leaf has been worn by peasant and knight on the falchion arm. In Erin's isle especially, the holy trefoil's charm has been always respected as an unfailing protection against evil influences, which fact the spray in the cap of the workman, or in the bosom of the cotter's wife, fully attests. Whatever real virtue the red clover possesses for counteracting the scrofulous disposition, and as a hopeful antidote to cancer, must reside in its highly-elaborated lime, silica, and other earthy salts. Such an experience is by no means new. Sir Spencer Wells, twenty years ago, recorded some cases of confirmed cancer cured by the taking of powdered oystershells; and triturated egg-shells have been found to prove similarly useful. If the moorlands in the north of England, and in some parts of Ireland, are turned up for the first time, and strewed with lime, white clover springs up there in abundance.

The word clover is a corruption of the Latin clava, "a club"; and the clubs on our playing-cards are representations of clover leaves. In France the same black suit is called "trefle." The meadow clover can only be fertilised by bees with a long proboscis; but the burgling bumble will often purloin honey by biting a hole through the side of the flower. "Living in clover" alludes to cattle sent to feed in rich pastures, and may be put on a floral par with "laid up in lavender." A sworn foe to the purple clover cultivated by farmers is the dodder (cuscuta trifolii), a destructive vegetable parasite—"smiling, smooth, detested"—which strangles the crop in a crafty fashion, and which goes by the name of hellweed, or "devil's guts." It lies in ambush like a pigmy field octopus, with deadly suckers for draining the sap of its victims. These it mats together in its wiry, sinuous coils, and chokes relentlessly by the acre. Nevertheless, the petty garotter, like a toad, "ugly and venomous, wears yet a precious jewel in its head." "If boiled," says Hill, "with a little ginger, the dodder in decoction works briskly as a purge. Also the thievish herb when bruised and applied externally to scrofulous tumours is an excellent remedy." In common with many other obnoxious creatures it seems to possess certain special healing virtues. Similarly, from the common bed bug (cimex lectularius) a tincture is prepared by American physicians which is powerfully emmenagogue. And cockroach-tea, made from the disgusting blatta, is a remedy recognised by the faculty in Russia for curing chronic Bright's disease. The dodder is sometimes called Red-tangle and Lady's-laces.

### COMFREY.

The comfrey of our river banks and moist, watery places is the consound, or knit-back, or bone-set of rustics; and the old symphytum of Dioscorides. It has derived these names from the consolidating and vulnerary qualities attributed to the plant. "Quia tanta præstantia est," says Pliny, "ut carnes, dum coquuntur, conglutinet addita; unde nomen"—"the roots be so glutinatine that they will soder, or glew together meat that is chopt in pieces, seething in a pot, and make it into one lump; the same bruysed, and layd in the manner of a plaister, doth heale all fresh and green wounds." These roots are inodorous, sweet, and sticky. In Angora a a sort of glue is obtained therefrom, which is used for spinning the famous fleeces of that country. The plant belongs to the forage tribe, and is conspicuous by its height of from one to two feet, its large, rough leaves, which provoke itching when handled, and its drooping white or purple flowers, growing on short stalks. Formerly the leaves were employed for giving a flavour to cakes and panada; also the whole plant beaten to a cataplasm, and applied hot as a poultice, was thought to be most excellent for healing pain in any tender, inflamed, or suppurating part. It was likewise applied to raw, indolent ulcers, being a glutinous, astringent, and admirable vulnerary. Pauli recommended it for broken bones; and, externally, for wounds of the nerves, tendons, and arteries. More recent physicians have declared that the powdered root (which, when broken, is white within, and full of a slimy juice), if dissolved in water to a mucilage, is far from being contemptible for bleedings, fractures, and luxations, whilst it hastens the callus of bones under repair. A modern medicinal tincture is made from the fresh root-stock, with proof spirit of wine; and ten drops of this should be taken three or four times a day, with a table-spoonful of cold water. The Prophet Isaiah may have had some foreknowledge of comfrey when predicting that in times to come the heart should rejoice, and the bones flourish like a herb. Chemically, the most important part of the plant is the mucilage. It also contains tannin, asparagin, sugar, and starch-granules. French nurses treat cracked nipples by applying a hollow section of the fresh root over the sore organ; and a decoction of the root, made by boiling from two to four drams in a pint of water, is given for bleeding from the lungs or bladder. About a century ago the prickly comfrey—a variety of our consound—was naturalised in this country from the Caucasus, and it has since proved itself amazingly productive to farmers. When cultivated it will produce six crops in the year, and it is permanent after once getting a foothold. Moreover, it is both preventive and curative of foot and mouth disease in cattle. Therefore it has become an excellent forage plant in many districts of England and Wales. It bears flowers of a rich blue colour. The name consound, owned by our common comfrey, was also given to the daisy and the bugle in the middle ages. It joyeth—says Gerarde—in watery ditches, in fat and fruitful meadows. A salve made from the fresh herb certainly helps to conjoin bruised and broken parts. It well merits the motto for its box: "Behold! how good and pleasant a thing it is to dwell together in unity! It is like the precious ointment which ran down Aaron's beard!"

